# The First Evaluation of Remote Magnetic Navigation-Guided Pediatric Ventricular Arrhythmia Ablation

**DOI:** 10.1007/s00246-022-02900-5

**Published:** 2022-04-29

**Authors:** Anna M. E. Noten, Nawin L. Ramdat Misier, Janneke A. E. Kammeraad, Sip Wijchers, Ingrid M. Van Beynum, Michiel Dalinghaus, Thomas B. Krasemann, Sing-Chien Yap, Natasja M. S. de Groot, Tamas Szili-Torok

**Affiliations:** 1 Department of Cardiology, Erasmus MC, University Medical Center, Rotterdam, The Netherlands; 2 Department of Pediatric Cardiology, Erasmus MC, University Medical Center, Rotterdam, The Netherlands; 3 Department of Clinical Electrophysiology, Thoraxcenter, Erasmus MC, Postbus 2040, 3000 CA Rotterdam, The Netherlands

**Keywords:** Catheter ablation, Remote magnetic navigation, Radiofrequency ablation, Ventricular tachycardia, Premature ventricular complex, Ventricular arrhythmia, Pediatrics

## Abstract

Catheter ablation (CA) is an important treatment option for ventricular arrhythmias (VA) in pediatric cardiology. Currently, various CA techniques are available, including remote magnetic navigation (RMN)-guided radiofrequency (RF) ablation. However, no studies evaluate RMN-guided ablative therapy outcomes in children with VA yet. This study aimed to compare procedural and long-term outcomes between RMN-guided and manual (MAN)-guided VA ablation in children. This single-center, retrospective study included all CA procedures for VA performed in children with or without structural heart disease from 2008 until 2020. Two study groups were defined by CA technique: RMN or MAN. Primary outcome was recurrence of VA. Baseline clinical, procedural and safety data were also evaluated. This study included 22 patients, who underwent 30 procedures, with a median age of 15 (IQR 14–17; range 1–17) years and a mean weight of 57 ± 20 kg. In total, 14 procedures were performed using RMN and 16 using MAN (22 first and 8 redo procedures). Regarding first procedures, recurrence rates were significantly lower in RMN compared to MAN (20% versus 67%, *P* = 0.029), at a mean follow-up of 5.2 ± 3.0 years. Moreover, fluoroscopy dosages were significantly lower in RMN compared to MAN [20 (IQR 14–54) versus 48 (IQR 38–62) mGy, *P* = 0.043]. In total, 20 patients (91%) were free of VA following their final ablation procedure. This is the first study to investigate the use of RMN in pediatric VA ablation. RMN showed improved outcomes compared to MAN, resulting in lower VA recurrence and reduced fluoroscopy exposure.

## Introduction

Ventricular tachycardia (VT) and premature ventricular contractions (PVC) are less common rhythm disorders than supraventricular arrhythmias in children with normal cardiac anatomy [1, 2]. Generally originating with an idiopathic etiology, they usually have a benign course with spontaneous resolvement of VA. However, in some cases untreated idiopathic ventricular arrhythmia (VA) can be associated with impaired quality of life, substantial morbidity—including hemodynamic deterioration and ventricular dysfunction—and rarely mortality [3–7].

Catheter ablation (CA) is an important treatment option for children who have failed medical treatment of VA or who suffer from a VA resulting in LV dysfunction [8]. Moreover, CA presents the possibility of complete elimination of the rhythm disorder. In children, the reported experience of catheter ablation of VA is limited, highlighting the scarcity of VA ablative therapy in this population [5, 9–15]. Besides, acute and long-term success rates are both suboptimal and variable between centers. In some studies, up to one third of children experience arrhythmia recurrence following their initial ablation [5, 9–15]. Failure of CA and high recurrence rates may be explained by the lack of specifically designed small curved catheters for these patients, the non-inducibility of the tachycardia at procedure baseline, and to inherent technical challenges of the ablation site—including unstable catheter-tissue contact and close proximity to regions at risk for complications.

Currently, various CA techniques are utilized in the adult population. In children, manual (MAN)-guided radiofrequency (RF) ablation is still the most frequently used technique for VA ablation [8]. In adults, remote magnetic navigation (RMN)-guided RF ablation of VT has shown to be superior to manual-guided RF ablation in patients without structural heart disease [16–18]. Moreover, various studies reported significant advantages of RMN-guided ablation of supraventricular tachyarrhythmias (SVT) in children and adults with congenital heart defect (CHD) [19–22]. At present, no study has systematically evaluated the use of RMN guidance in pediatric VA ablation. Thus, we present the first-in-human investigation of this technique from a single high-volume CA center. This study aims to evaluate and compare procedural data and long-term outcomes between RMN- and manual-guided VA ablation in children.

## Methods

### Study Design and Population

This single-center, retrospective study, investigated all consecutive CA procedures using RMN or manual-guided RF techniques in children (aged < 18 years) as treatment of documented VA (including PVC or VT) between the 1st of January 2008 until 31th of December 2020. We excluded patients in whom cross-over between CA techniques occurred. Outcomes were compared between two groups, based on the ablation technique used: conventional manual-guided RF ablation (MAN) or RMN-guided RF ablation (RMN). All patients scheduled for VA ablation were distributed from the waiting list based on availability to the RMN-equipped or the conventional EP laboratory. Accordingly, ablation was performed either using RMN or using conventional MAN. The procedures were performed over the entire study duration by the same senior electrophysiologist group with the assistance of fellows, trained for manual catheter navigation as well as for RMN. The attending physicians performed RMN and MAN procedures in equal distribution. The primary endpoint for PVC ablation was > 90% reduction in PVC burden in comparison to baseline during long-term follow-up as measured by Holter continuous rhythm observation. The primary endpoint for VT ablation was absence of recurrence of sustained VT during long-term follow-up. We also analyzed the following secondary endpoints: procedural parameters (including fluoroscopy dosages), acute procedure success, redo procedure rates, and complication rates. The study protocol conforms to the ethical guidelines of the 1975 Declaration of Helsinki. The local medical ethics committee determined this study was not subject to the Dutch Medical Research Involving Human Subjects Act (WMO). The need for individual consent was waived by the local medical ethical committee.

### Data Collection

Baseline demographic and clinical characteristics were collected from the institutional electronic patient dossier. Procedural data were derived both from the electronic medical files, as well as from the electronic procedural log files recorded with the EP-workmate (St. Jude Medical Inc., St. Paul, MN, USA), EnSite NavX (St. Jude Medical Inc., St. Paul, MN, USA), CARTO (Biosense Webster Inc., Diamond Bar, CA, USA) and Odyssey Cinema (Stereotaxis Inc., St. Louis MO, USA) systems. All patient information was de-identified.

### Definitions

Total procedure time was defined as the time from first puncture until the removal of sheaths. Major complications were permanent second- or third-degree atrioventricular block, cardiac tamponade, hemorrhagic shock, stroke, and procedure-related death. Minor complications were access site complications, temporary bundle branch block or AV block, pericardial effusion not requiring intervention. We considered as access site complications: bleeding complications or local vascular wall damage which required surgical intervention or intervention by radiologist, prolonged hospitalization and/or Hb drop of > 1.8 mmol/L.

### Procedural Protocol

All antiarrhythmic drugs were discontinued for > 5 half-lives before the procedure, except amiodarone. Procedures were performed under general anesthesia as per standard approach. Occasionally procedures were performed only with local anesthetic, based on operator preference when there was lack of PVC during sedation and patient/parent agreement. Vascular access was attained with placement of sheaths and diagnostic catheters in left and/or right femoral vein and/or artery, with sheath sizes and catheter types varying based on operator preference. Left-sided targets were approached by the retrograde aortic route. Three‐dimensional electro-anatomic mapping (EAM) was performed in all patients using the EnSite NavX (St. Jude Medical Inc., St. Paul, MN, USA) or the CARTO (Biosense Webster Inc., Diamond Bar, CA, USA) systems. Ablation was performed using the following radiofrequency settings in RMN: RV and RVOT: 45–50 W, flow 20 mL/min, maximum 43 °C; aortic cusp: 20 W gradually increasing to 45–50 W, flow 30 mL/min, maximum 43 °C; left ventricle (LV): 50–55 W, 30 ml/min, maximum 43 °C. In MAN the following settings were used: RV and RVOT: 25–40 W, flow 20 mL/min, maximum 43 °C; aortic cusp: 10 W gradually increasing to 40 W, flow 30 mL/min, maximum 43 °C; LV: 25–45 W, flow 30 mL/min, maximum 43 °C. The Navistar RMT ThermoCool (Biosense Webster, Diamond Bar, CA, USA) ablation catheter was used in all RMN-guided procedures. In the MAN group the following ablation catheters were used: Navistar ThermoCool (Biosense Webster, Diamond Bar, CA, USA), Blazer (Boston Scientific, Marlborough, MA, USA), ThermoCool SmartTouch (Biosense Webster, Diamond Bar, CA, USA) and TactiCath EndoSense (St. Jude Medical Inc., St. Paul, MN, USA). Contact force sensing catheters were introduced in our center in 2013.

### Ablation Target

In patients with PVCs, the ablation target was the site of the earliest activation during activation mapping and/or the site of identical pace mapping. Acute success was defined as complete suppression of PVCs in the operating theater and during continuous rhythm monitoring during in-hospital stay. Regarding VT, if not incessant, VT was induced by programmed electrical stimulation (PES) and activation or entrainment mapping was performed if VT was hemodynamically tolerable, to identify the mechanism and to locate site of origin and/or critical isthmuses and exit sites. In patients with enhanced automaticity and triggered activity mechanisms, the site of the earliest activation during activation mapping and/or the site of identical pace mapping was the target of ablation. In patients with reentry mechanisms (including patients with fascicular or scar-related VT), the main target was the critical isthmus using conventional diagnostic criteria (i.e. mid-diastolic potentials). Another target was the exit site of the VT circuit identified during activation mapping or pace mapping. In patients with VT, acute success was defined as non-inducibility of any VT using PES at the end of procedure. Testing with isoprenaline at the end of procedure was performed based on operator’s preference. A standard of 30 min waiting time was applied to all procedures.

### Follow-Up

Patients were seen at our outpatient clinic at 2 months after the procedure and subsequently on annual check-ups. When patients experienced symptoms, they were seen more frequently as determined by the treating physician. Pre-procedural PVC burden (number of PVCs/total number of beats) was collected in all patients, and was used as the reference value. Long-term success was assessed by Holter rhythm monitoring performed 3–6 months after catheter ablation in all patients, except in those with an implanted device (2 patients with ICD, 1 patient with ILR). Holter rhythm monitoring was performed more frequently if patients experienced symptoms during follow-up. Regarding PVC, long-term success was defined as a reduction of at least 90% of the PVC burden compared to the pre-procedural assessment. Regarding patients diagnosed with VTs, long-term success was regarded when a patient had no recurrences of sustained VT recorded on either 12-lead ECG, Holter rhythm monitoring, ILR, pacemaker or ICD.

### Statistical Analysis

Normality was assessed by the Kolmogorov–Smirnov test, or when appropriate, Shapiro–Wilk test. Mean and standard deviation (SD) were calculated for normally distributed continuous variables. Median, interquartile range (IQR) and ranges were computed for continuous variables with non-normal distribution. Descriptive statistics for categorical data were expressed in absolute numbers and percentages. Normal distributed continuous variables were analyzed using the Student’s *T*-test. For continuous variables with non-normal distributions, the Mann–Whitney *U* test was used. For comparing frequencies, the Chi-square test was used, or, when appropriate, Fisher's exact test. A 2-sided *P*-value of < 0.05 was considered significant. Data were analyzed using SPSS 26.0 (SPSS Inc., Chicago, IL, USA).

## Results

**Fig. 1:**
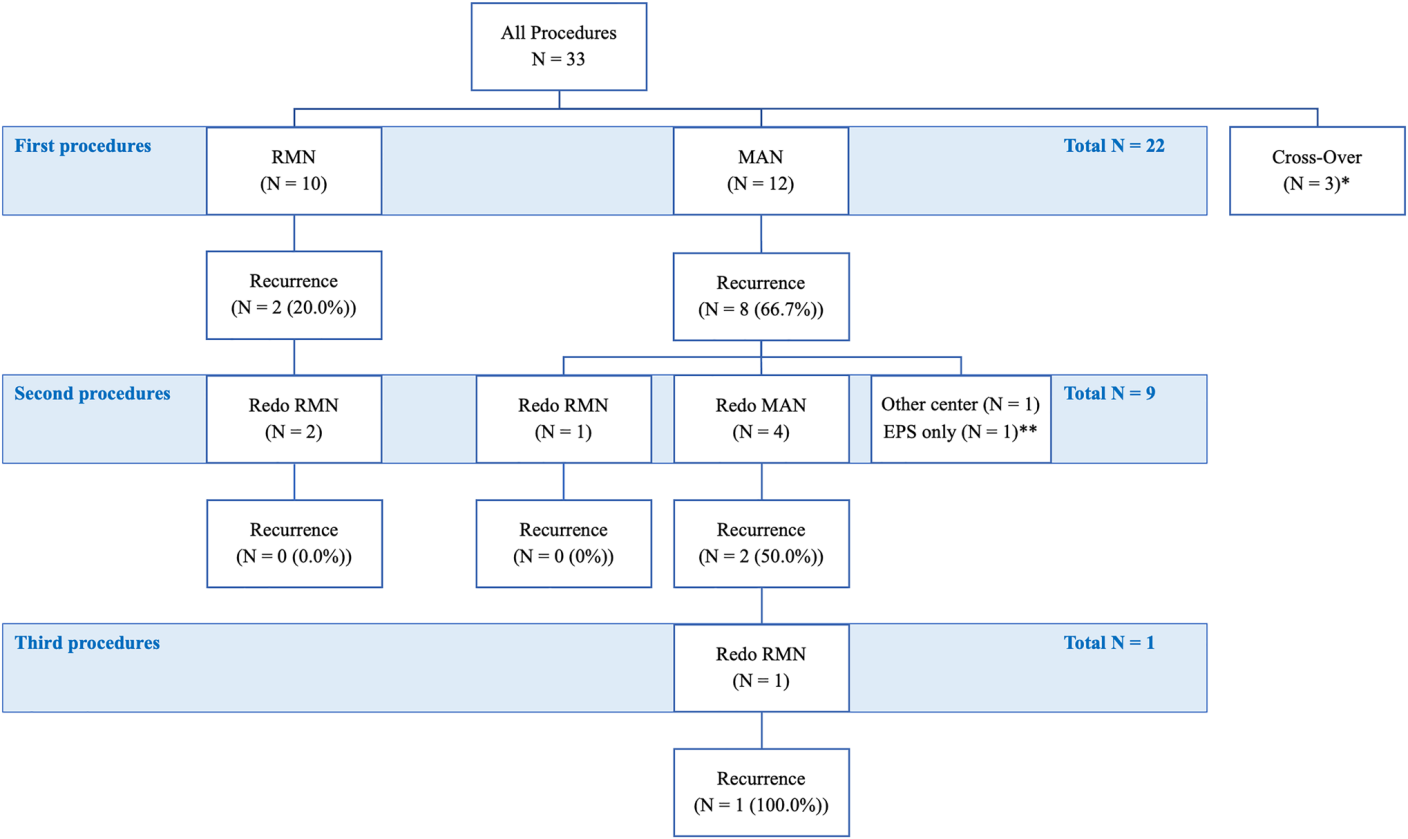
First and redo procedures. Schematical figure of all first and redo procedures, including recurrence rates. *In three patients a cross-over between techniques occurred. In two cases there was switch from MAN ablation to cryoablation; in one case there was a switch from RMN to MAN. **Of the nine patients who underwent a redo procedure, one was performed in another center and one was an electrophysiological study (EPS) only. *EPS electrophysiological study, MAN manual-guided radiofrequency ablation, RMN remote magnetic navigation-guided radiofrequency ablation*

In total, 33 VT and/or PVC ablation procedures were performed within the mentioned time frame. A cross-over between techniques occurred in 3 procedures, which were excluded (in two cases switch from MAN to cryoablation; in one case switch from RMN to MAN). Data from the remaining 30 procedures, performed in 22 patients were analyzed. These were 22 (73%) first procedures and 8 (27%) redo procedures. With respect to the first procedures, 10 patients (46%) were treated with RMN, whereas 12 (55%) were treated with the MAN technique. Regarding the redo procedures, 4 (50%) were performed with RMN and 4 (50%) with MAN (Fig. 1).

### Baseline Demographic and Clinical Data

Baseline demographic and clinical data are presented in Table 1. Patients had a median age of 15 (IQR 14–17; range 1–17) years, with a mean weight of 57 ± 20 (range 16–95) kilograms. A minority was female (36%). Fifteen patients (68%) had no structural heart disease, whereas 3 patients (14%) had a dilated cardiomyopathy (DCM) and 4 patients (18%) had a CHD. In the patients with CHD the main defects were: (1) restrictive ventricular septal defect (VSD) (uncorrected), (2) mild native Ebstein’s anomaly (uncorrected) (3) double outlet right ventricle (DORV) with transposition of the great arteries (TGA), ASD II, VSD and valvular and supravalvular pulmonalis stenosis, for which initially a Blalock Taussig shunt and later a Rastelli operation with a pulmonalis homograft was performed (4) TGA and VSD for which arterial switch operation and VSD closure was performed. Most patients had a normal left ventricular (LV) ejection fraction (EF) and right ventricular (RV) EF on TTE (77% and 96%, respectively), with normal LV and RV dimensions (82% and 96%, respectively). CHD was more frequently present in MAN compared to RMN (33% versus 0%, *P* = 0.044).

**Table 1:** Baseline demographic and clinical data

	RMN *N* = 10	MAN *N* = 12	Total *N* = 22	*P*-value
Female	4 (40%)	4 (33%)	8 (36%)	0.75
Age (year)	16 (14–17)	14 (12–17)	15 (14–17)	0.38
Weight (kg)	62 ± 22	53 ± 18	57 ± 20	0.31
No structural HD	8 (80%)	5 (42%)	15 (68%)	0.28
DCM	2 (20%)	1 (8%)	3 (14%)	0.43
CHD	0 (0%)	4 (33%)	4 (18%)	0.044
ICD	1 (10%)	1 (8%)	2 (9%)	0.89
Family history of sudden death	1 (10%)	1 (8%)	2 (9%)	0.89
LVEF normal	7 (70%)	10 (83%)	17 (77%)	0.46
LVEF decreased	2 (20%)	1 (8%)	3 (14%)	0.43
LVEF poor (< 30%)	1 (10%)	1 (8%)	2 (9%)	0.89
RVEF normal	10 (100%)	11 (92%)	21 (96%)	0.35
RVEF decreased	0 (0%)	1 (8%)	1 (5%)	0.35
Indication				
Palpitations	9 (90%)	11 (92%)	20 (91%)	0.89
Syncope	2 (20%)	5 (42%)	7 (32%)	0.28
PVC	2 (20%)	5 (42%)	7 (32%)	0.19
VT	5 (50%)	3 (25%)	8 (36%)	0.19
Both VT and PVC	3 (30%)	4 (33%)	7 (32%)	0.87
PVC burden (%)	37 (10–48)	31 (24–42)	36 (17–45)	0.94
Medication				
None	3 (30%)	3 (25%)	6 (27%)	0.79
Beta-blocker	2 (20%)	4 (33%)	6 (27%)	0.48
Sotalol	1 (10%)	0 (0%)	1 (5%)	0.26
Digoxine	0 (0%)	1 (8%)	1 (5%)	0.35
Amiodarone	1 (10%)	2 (17%)	3 (14%)	0.65
Verapamil	1 (10%)	4 (33%)	5 (23%)	0.19
Flecainide	3 (30%)	0 (0%)	3 (14%)	0.041

*BMI* body mass index, *CHD* congenital heart defect, *DCM* dilated cardiomyopathy, *HD* heart disease, *ICD* implantable cardioverter defibrillator, *LV* left ventricle, *LVEF* left ventricular ejection fraction, *MAN* manual-guided radiofrequency ablation, *PVC* premature ventricular complex, *RMN* remote magnetic navigation-guided radiofrequency ablation, *RV* right ventricle, *RVEF* right ventricular ejection fraction, *VT* ventricular tachycardia

Palpitations were the most apparent symptom and observed in 91% of patients. Seven patients (32%) had syncope. In all patients, there was documentation of either PVC (32%), VT (36%) or both (32%). In patients with PVC’s, the median preprocedural PVC burden was 36% (IQR 17–45%, range 5–52%). Baseline demographic and clinical data were comparable between groups.

### Procedural Data

**Fig. 2:**
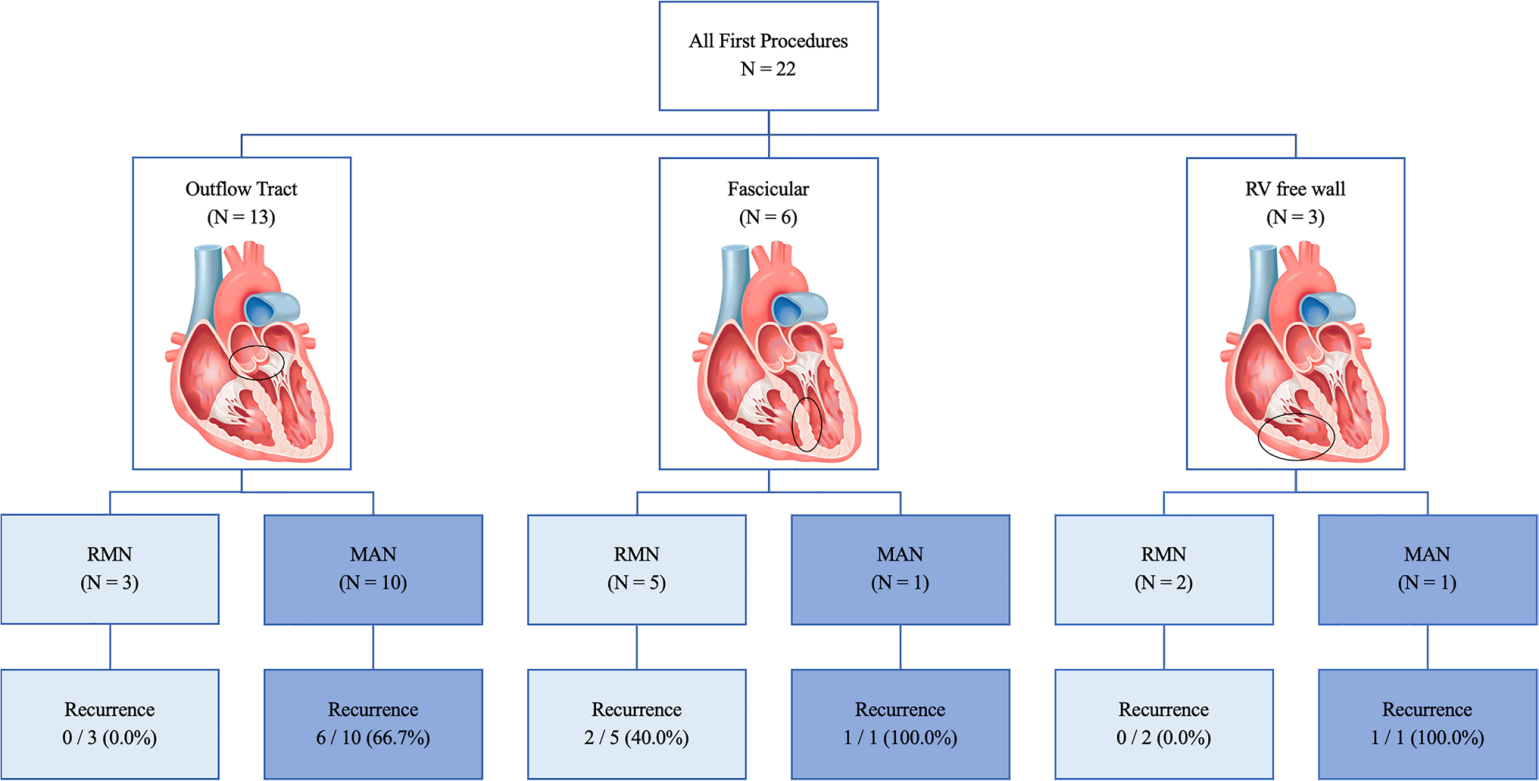
Recurrence rates for specific VT targets. This figure displays the distribution of the VT targets of patients included in the study—outflow tract VA, fascicular VA and RV free wall VA—and displays the recurrence rates for the two ablation techniques per target. *MAN manual-guided radiofrequency ablation, RMN remote magnetic navigation-guided radiofrequency ablation*

In all RMN patients, PVC/VT was inducible at procedure baseline and subsequently activation mapping performed. In MAN the inducibility at baseline was 83%, which was comparable between groups (*P* = 0.18). More fascicular VT’s were treated with RMN compared to MAN (50% versus 8%, respectively, *P* = 0.029). Outflow tract PVC/VT’s were less frequently treated with RMN compared to MAN (30% versus 83%, respectively, *P* = 0.003). Most outflow tract targets were located in the RVOT [RVOT 11 (92%), LVOT 1 (8%), Aortic Cusp 1 (8%)]. In all patients with fascicular VT, the posterior fascicle was ablated (6 out of 6 (100%). Other LV/RV targets of VA were found in 3 patients (14%), which was equally distributed between groups (*P* = 0.19). These included: (1) RV free wall anterior just below tricuspid valve, (2) RV free wall antero-lateral just below tricuspid valve and (3) RV septal infero-posterior involving the Purkinje network. The distribution of targets and treatment modalities is presented in Fig. 2. Of the patients with CHD, two had PVC’s originating from the RVOT and two had a fascicular VT. In this cohort, no patient with structural heart disease had scar-related VT.

Procedure and ablation times were comparable between groups [134 (IQR 107–199) min versus 128 (IQR 103–153) min, *P* = 0.72 and 305 (IQR 13–786) seconds versus 842 (IQR 220–1233) s, *P* = 0.21; for RMN versus MAN, respectively]. Fluoroscopy dosages were significantly lower in RMN compared to MAN (20 (IQR 14–54) mGy versus 48 (IQR 38–62) mGy, *P* = 0.043). In 96% of patients acute success was observed, which was comparable between groups (90% versus 100%, *P* = 0.26) (Table 2).

**Table 2:** Procedural parameters

	RMN *N* = 10	MAN *N* = 12	Total *N* = 22	*P*-value
Approach—ride sided transvenous	5 (50%)	10 (83%)	15 (68%)	0.10
Approach—left-sided retrograde aortic	5 (50%)	2 (17%)	7 (32%)	0.10
VT/PVC inducibility at baseline	10 (100%)	10 (83%)	20 (91%)	0.18
Activation mapping	10 (100%)	10 (83%)	20 (91%)	0.18
Pace mapping	5 (50%)	9 (75%)	14 (64%)	0.23
Target				
Outflow tract	3 (30%)	10 (83%)	13 (59%)	0.011
Fascicular	5 (50%)	1 (8%)	6 (27%)	0.029
Other*	2 (20%)	1 (8%)	3 (14%)	0.43
Parameters				
Procedure time (min)	134 (107–199)	128 (103–153)	134 (105–148)	0.72
Fluoroscopy dosage (mGy)	20 (14–54)	48 (38–62)	41 (17–55)	0.043
Ablation time (s)	305 (13–786)	842 (220–1233)	448 (31–1063)	0.21
Application number	24 (8–33)	18 (4–26)	24 (8–28)	0.41
Acute success				
Acute success	9 (90%)	12 (100%)	21 (96%)	0.26
PVC/VT termination	9 (90%)	12 (100%)	21 (96%)	0.26
Non-inducibility clinical PVC/VT	9 (90%)	12 (100%)	21 (96%)	0.26
Non-inducibility any arrhythmia	9 (90%)	10 (83%)	19 (86%)	0.65

*MAN* manual-guided radiofrequency ablation, *PVC* premature ventricular complex, *RMN* remote magnetic navigation-guided radiofrequency ablation, *VT* ventricular tachycardia

*Other locations of VT where: RV free wall anteriorly below tricuspid valve; RV free wall anterior-lateral below tricuspid valve; RV septal, inferior-posterior involving Purkinje network

### Long-term Outcome

We observed significantly fewer recurrences of VA in patients treated with RMN, compared to MAN (20% versus 67%, respectively, *P* = 0.029) at a mean follow-up time of 5.2 ± 3.0 years (Table 3 and Fig. 2). Follow-up time was comparable between groups (RMN: 4.6 ± 3.4 years versus MAN: 5.8 ± 2.5 years, *P* = 0.20). Two patients had a follow-up duration < 1 year because of transferral to another hospital (one RMN, one MAN). The redo procedure rate was higher in MAN group when compared to RMN, albeit not significant (RMN: 20% versus MAN: 58%, *P* = 0.070). Of the nine patients who underwent a redo procedure, one was performed in another center and another had no inducibility of any arrhythmia during the repeat procedure (i.e. no ablation was performed). The remaining 7 patients underwent 8 redo procedures in our center (Fig. 1). During all redo procedures the same target was ablated as during the first procedure, except for one patient where during the first procedure only the RVOT was treated and in the redo procedure both the RVOT and LVOT were ablated. In total, 20 patients (91%) were free of PVC/VT recurrence following their final ablation procedure.

**Table 3:** Long-term outcomes

	RMN *N* = 10	MAN *N* = 12	Total *N* = 22	*P*-value
PVC reduction > 90%*	5/5 (100%)	5/10 (56%)	10/14 (71%)	0.08
VA Recurrence	2 (20%)	8 (67%)	10 (46%)	0.029
Time to Recurrence (months)	0 (0–0)	2 (1–9)	2 (0–8)	0.38
Redo procedure	2 (20%)	7 (58%)	9 (41%)	0.07
Redo procedure number	1 (1–1)	1 (1–2)	1 (1–1)	0.08

*MAN manual-guided radiofrequency ablation, PVC premature ventricular complex, RMN remote magnetic navigation-guided radiofrequency ablation, VA ventricular arrhythmia, VT ventricular tachycardia*

*If PVC were present pre-procedural

### Adverse Events

We observed no major complications and one minor complication in a patient treated with RMN who had temporary AV block, that resolved completely after the procedure (RMN 1 (10%) versus MAN 0 (0%), *P* = 0.26). One patient from the MAN group died 5 year after the ablation following an out-of-hospital cardiac arrest for which resuscitation was performed. This patient had a double outlet right ventricle and had no ICD implanted. The cause of the circulatory arrest was unknown. The patient had no recurrence of sustained VT during regular annular check-ups.

## Discussion

This is the first study to describe the use of RMN-guided RF ablation for pediatric PVC and VT ablation in a unique single-center population. The main finding of this study is that RMN is a feasible ablation technique for these arrhythmias in children and has improved long-term efficacy when compared to manual RF ablation.

### Efficacy

Significantly fewer recurrences were observed in patients treated with RMN compared to MAN (20% versus 67%) at a mean follow-up of more than 5 years. The better outcome of patients treated with RMN can be attributed to some of the technical advantages of its design [23]. RMN catheters are not limited by fixed curves, have a greater flexibility and make more uniform contact, all of which contribute to improved lesion formation [23]. Magnetic-guided ablation by itself aids to achieve more adequate lesion formation by enhanced catheter stability and consequently improved contact with the myocardial wall [24]. This is of critical importance in cardiac regions with greater wall motion excursion such as the ventricle. A magnetic catheter also appears to produce less catheter-induced-ectopy, which is particularly important in PVC ablation when a mechanical PVC could cause the operator to ablate the wrong target [25]. The RMN-guided catheter’s position can be stored and reapplied, which presents the ability to return precisely to critical locations to either assess delivery impact or deliver additional therapy. RMN facilitates titration of CF between the catheter and the myocardial tissue [23, 26]. Therefore, RMN avoids excessive catheter indentations on cardiac wall that sometimes are unintentionally applied during manual manipulation of conventional catheters, which may eventually be responsible for cardiac perforation and tamponade [26, 27]. These technical characteristics of RMN-guided catheter ablation are extremely beneficial in a small anatomy and, in addition, in treatment of targets at locations with marked wall excursions such as the ventricle. At present, ablative therapy remains reserved for highly symptomatic children who have failed medical treatment and/or established LV dysfunction [8]. The current study illustrated that RMN-guided CA procedures in this population are highly effective and safe, resulting in complete elimination of the rhythm disorder.

### Fascicular VT Versus Outflow Tract VT

Significantly more fascicular VT were treated in the RMN group than in the manual group (50.0% versus 8.3%). This might have biased the outcomes, however, it is unlikely that the observed differences in outcomes—including fluoroscopic times and recurrence rates—are a result of this. Previously, Wu et al. showed that acute and long-term outcome did not differ between manual ablation of fascicular and nonfascicular VT in pediatric patients [28]. Similarly, Collins et al. reported no difference in initial success—with and without an intention to treat analysis – and recurrence rates between left-sided fascicular and nonfascicular VT ablation in pediatrics [5]. In addition, inclusion of more outflow tract VT in RMN group may even accentuate the improved performance of RMN-guided ablation, as its substrate may particularly benefit from the extra stability and flexibility provided by RMN, as shown in adults by Kawamura et al. [29].

### Fluoroscopy Exposure

Besides improvement in clinical outcomes, advances in catheter ablation should strive to reduce radiation exposure, especially in pediatrics, as its effects are substantial for them. Compared to adults, children are ten times more vulnerable to the induction of cancer by external radiation exposure [30]. In our study, both techniques had low fluoroscopy dosages, but the lowest values were observed in RMN. This significant improvement in fluoroscopy exposure can be attributed to several factors. We hypothesize that the enhanced stability and flexibility enables faster access to the ablation site. Moreover, improved catheter stability causes less concern for catheter dislodgement resulting in less fluoroscopic confirmation of tip location. In addition, the atraumatic design of the catheter may result in more physician comfort maneuvring without continuous fluoroscopy. Collins et al. observed in an international multicentre study, including 152 pediatric patients, similar fluoroscopy times between ablation of fascicular and nonfascicular left VT (24 versus 28 min), comparable to the fluoroscopy times seen in patients treated with MAN ablation in this study [5]. Hence, the observed difference in our study is most likely a consequence of the ablation method, and to a lesser extent of the ablation indication. Nowadays, the utilization of 3D electroanatomic mapping systems and intracardiac echocardiography (ICE) systems allows for ablation procedures with limited or even without any fluoroscopy. Several studies observed that fluoroless ablation can be safely used in pediatric patients [31–33], also with respect to left-sided targets. Given the retrospective nature and inclusion period of our cohort, fluoroless ablation was not attempted in the current study cohort. However, in our opinion it is the current most favorable approach, especially when ablating the young patient.

### Limitations

Due to the retrospective design of the study, its results are subjected to typical bias and limitation of retrospective research. Given the scarcity of VA ablative therapy during childhood, we had to search for a balance between inclusion period and numbers. In addition, this emphasizes the rarity of CA for ventricular rhythm disorders in children. The study population consisted of 7 patients with native structural heart disease (32%). However, as none had a scar-related VT, its impact on ablation technique outcomes is expected to be minimal. Despite these limitations, we believe this study presents an important first comparison of CA techniques in this particular population. Further prospective or randomized studies with larger and more homogenous groups, such as the OSCAPED registry, are required to confirm the observed outcomes, though difficult given the uncommonness of VA in children.

In conclusion, RMN is an effective and safe catheter ablation technique for the elimination of pediatric PVC and VT, resulting in significantly lower recurrence rates when compared to manual ablation, in addition to a low fluoroscopy exposure.

## Abbreviations

CA: Catheter ablation
CHD: Congenital heart defect
DAP: Dose area product
DCM: Dilated cardiomyopathy
EAM: Electro-anatomic mapping
ECG: Electrocardiogram
EF: Ejection fraction
ICD: Implantable cardioverter defibrillator
ILR: Implantable loop recorder
LV: Left ventricle
MAN: Manual ablation
OR: Operation room
PES: Programmed electrical stimulation
PVC: Premature ventricular tachycardia
RF: Radiofrequency
RMN: Remote magnetic navigation
RV: Right ventricle
VA: Ventricular arrhythmia
VT: Ventricular tachycardia

## References

[1] Roggen A, Pavlovic M, Pfammatter JP (2008). Frequency of spontaneous ventricular tachycardia in a pediatric population. Am J Cardiol.

[2] Iwamoto M, Niimura I, Shibata T, Yasui K, Takigiku K, Nishizawa T (2005). Long-term course and clinical characteristics of ventricular tachycardia detected in children by school-based heart disease screening. Circ J.

[3] Pfammatter JP, Paul T (1999). Idiopathic ventricular tachycardia in infancy and childhood: a multicenter study on clinical profile and outcome. Working Group on Dysrhythmias and Electrophysiology of the Association for European Pediatric Cardiology. J Am Coll Cardiol.

[4] Hasdemir C, Ulucan C, Yavuzgil O, Yuksel A, Kartal Y, Simsek E (2011). Tachycardia-induced cardiomyopathy in patients with idiopathic ventricular arrhythmias: the incidence, clinical and electrophysiologic characteristics, and the predictors. J Cardiovasc Electrophysiol.

[5] Collins KK, Schaffer MS, Liberman L, Saarel E, Knecht M, Tanel RE (2013). Fascicular and nonfascicular left ventricular tachycardias in the young: an international multicenter study. J Cardiovasc Electrophysiol.

[6] Noda T, Shimizu W, Taguchi A, Aiba T, Satomi K, Suyama K (2005). Malignant entity of idiopathic ventricular fibrillation and polymorphic ventricular tachycardia initiated by premature extrasystoles originating from the right ventricular outflow tract. J Am Coll Cardiol.

[7] Bertels RA, Harteveld LM, Filippini LH, Clur SA, Blom NA (2017). Left ventricular dysfunction is associated with frequent premature ventricular complexes and asymptomatic ventricular tachycardia in children. Europace.

[8] Cronin EM, Bogun FM, Maury P, Peichl P, Chen M, Namboodiri N (2019). 2019 HRS/EHRA/APHRS/LAHRS expert consensus statement on catheter ablation of ventricular arrhythmias. Europace.

[9] Akdeniz C, Gul EE, Celik N, Karacan M, Tuzcu V (2016). Catheter ablation of idiopathic right ventricular arrhythmias in children with limited fluoroscopy. J Interv Card Electrophysiol.

[10] Krause U, Paul T, Bella PD, Gulletta S, Gebauer RA, Paech C, et al (2020) Pediatric catheter ablation at the beginning of the 21st century: results from the European Multicenter Pediatric Catheter Ablation Registry 'EUROPA'. Europace 10.1093/europace/euaa325 33227133

[11] Li XM, Jiang H, Li YH, Zhang Y, Liu HJ, Ge HY (2016). Effectiveness of radiofrequency catheter ablation of outflow tract ventricular arrhythmias in children and adolescents. Pediatr Cardiol.

[12] Schneider HE, Kriebel T, Jung K, Gravenhorst VD, Paul T (2010). Catheter ablation of idiopathic left and right ventricular tachycardias in the pediatric population using noncontact mapping. Heart Rhythm.

[13] Morwood JG, Triedman JK, Berul CI, Khairy P, Alexander ME, Cecchin F (2004). Radiofrequency catheter ablation of ventricular tachycardia in children and young adults with congenital heart disease. Heart Rhythm.

[14] Koca S, Akdeniz C, Karacan M, Tuzcu V (2019). Catheter ablation of left posterior fascicular ventricular tachycardia in children with limited fluoroscopy exposure. Cardiol Young.

[15] Chiu SN, Wu WL, Lu CW, Wu KL, Tseng WC, Lin MT (2017). Special electrophysiological characteristics of pediatric idiopathic ventricular tachycardia. Int J Cardiol.

[16] Szili-Torok T, Schwagten B, Akca F, Bauernfeind T, Abkenari LD, Haitsma D (2012). Catheter ablation of ventricular tachycardias using remote magnetic navigation: a consecutive case–control study. J Cardiovasc Electrophysiol.

[17] Akca F, Theuns DA, Abkenari LD, de Groot NM, Jordaens L, Szili-Torok T (2013). Outcomes of repeat catheter ablation using magnetic navigation or conventional ablation. Europace.

[18] Hendriks AA, Akca F, Dabiri Abkenari L, Khan M, Bhagwandien R, Yap SC (2015). Safety and clinical outcome of catheter ablation of ventricular arrhythmias using contact force sensing: consecutive case series. J Cardiovasc Electrophysiol.

[19] Schwagten B, Jordaens L, Witsenburg M, Duplessis F, Thornton A, van Belle Y (2009). Initial experience with catheter ablation using remote magnetic navigation in adults with complex congenital heart disease and in small children. Pacing Clin Electrophysiol.

[20] Ueda A, Suman-Horduna I, Mantziari L, Gujic M, Marchese P, Ho SY (2013). Contemporary outcomes of supraventricular tachycardia ablation in congenital heart disease: a single-center experience in 116 patients. Circ Arrhythm Electrophysiol.

[21] Akca F, Bauernfeind T, Witsenburg M, Dabiri Abkenari L, Cuypers JA, Roos-Hesselink JW (2012). Acute and long-term outcomes of catheter ablation using remote magnetic navigation in patients with congenital heart disease. Am J Cardiol.

[22] Roy K, Gomez-Pulido F, Ernst S (2016). Remote magnetic navigation for catheter ablation in patients with congenital heart disease: a review. J Cardiovasc Electrophysiol.

[23] Aagaard P, Natale A, Briceno D, Nakagawa H, Mohanty S, Gianni C (2016). Remote magnetic navigation: a focus on catheter ablation of ventricular arrhythmias. J Cardiovasc Electrophysiol.

[24] Bhaskaran A, Barry MA, Al Raisi SI, Chik W, Nguyen DT, Pouliopoulos J (2015). Magnetic guidance versus manual control: comparison of radiofrequency lesion dimensions and evaluation of the effect of heart wall motion in a myocardial phantom. J Interv Card Electrophysiol.

[25] Konstantinidou M, Koektuerk B, Wissner E, Schmidt B, Zerm T, Ouyang F (2011). Catheter ablation of right ventricular outflow tract tachycardia: a simplified remote-controlled approach. Europace.

[26] Bessiere F, Zikry C, Rivard L, Dyrda K, Khairy P (2018). Contact force with magnetic-guided catheter ablation. Europace.

[27] Noten AME, Hendriks AA, Yap SC, Mol D, Bhagwandien R, Wijchers S (2020). Contact feedback improves 1-year outcomes of remote magnetic navigation-guided ischemic ventricular tachycardia ablation. Int J Cardiol.

[28] Wu J, Chen Y, Ji W, Gu B, Shen J, Fu L (2020). Catheter ablation of ventricular tachycardia in the pediatric patients: a single-center experience. Pacing Clin Electrophysiol.

[29] Kawamura M, Scheinman MM, Tseng ZH, Lee BK, Marcus GM, Badhwar N (2017). Comparison of remote magnetic navigation ablation and manual ablation of idiopathic ventricular arrhythmia after failed manual ablation. J Interv Card Electrophysiol.

[30] Hall EJ (2002). Lessons we have learned from our children: cancer risks from diagnostic radiology. Pediatr Radiol.

[31] Žižek D, Antolič B, Prolič Kalinšek T, Štublar J, Kajdič N, Jelenc M (2021). Intracardiac echocardiography-guided transseptal puncture for fluoroless catheter ablation of left-sided tachycardias. J Interv Card Electrophysiol.

[32] Yazici M, Lakič N, Prolič Kalinšek T, Žižek D, Ažman Juvan K, Topalović M (2021). Fluoroless catheter ablation of accessory pathways in adult and pediatric patients: a single centre experience. Int J Cardiovasc Imaging.

[33] Clark BC, Sumihara K, Berul CI, Moak JP (2017). Off the pedal: Fluoroless transseptal puncture in pediatric supraventricular tachycardia ablation. Pacing Clin Electrophysiol.

